# Fuzzy Logic Tool to Forecast Soil Fertility in Nigeria

**DOI:** 10.1155/2018/3170816

**Published:** 2018-10-15

**Authors:** G. O. Ogunleye, S. G. Fashoto, Petros Mashwama, S. A. Arekete, O. M. Olaniyan, B. A. Omodunbi

**Affiliations:** ^1^Department of Computer Science, Federal University, Oye-Ekiti, Ekiti State, Nigeria; ^2^Department of Computer Science, University of Swaziland, Kwaluseni, Swaziland; ^3^Department of Computer Science, Redeemer's University, Ede, Osun State, Nigeria; ^4^Department of Computer Engineering, Federal University, Oye-Ekiti, Ekiti State, Nigeria

## Abstract

The soil is composed of several nutrients which are important for the effective growth of plants. Nitrogen, phosphorus, and potassium are micronutrients which are very important for plant growth. There have been several methods and soil tests developed to test the compositions of these nutrients in the soil. Interpreting the results gotten from such tests has been a herculean task for farmers. Employing the use of a soft computing method to interpret such result would be a noble idea. In this paper, we describe the use of fuzzy logic to interpret the values of nitrogen, phosphorus, and potassium (NPK) gotten from conventional soil test to know their levels in the soil and predict possible NPK inputs.

## 1. Introduction

Agriculture is a major sector in the Nigerian economy [1]. It accounts for the employment of up to 70% of Nigeria's population. Agriculture also contributes about 40% to the Gross Domestic Product (GDP) of Nigeria [2]. It also provides the country with food and raw materials for agro-allied industries.

Major crops grown in Nigeria include beans, sesame, cashew nuts, cassava, yam, cocoa beans, groundnut, gum arabic, kola nut, maize, millet, palm kernel, palm oil, plantain, rice, rubber, sorghum, soybeans, and yam. There have also been series of programs initiated by the government to improve the state of agriculture in Nigeria such as National Fadama I &II project, River Basin Development Authorities, Operation Feed the Nation, etc. All these methods adopted by various governments in Nigeria have not yielded good results so far. The government is now trying to diversifying the economy back to agriculture because in the past years attention has only been focusing on petroleum.

The fertility of the soil is determined by several factors such as light, soil composition, PH, nutrients, and climate change. These factors affect the constituents of the soil. It is necessary to know the constituents of the soil and over the years several methods have been developed and are in use to measure the soil constituents such as indicator methods, metal-electrode, glass-electrode methods, and semiconductor sensor methods for measuring soil PH, direct light indicator for measuring light intensity, litmus test, and a test probe to measure soil PH and a dry test to know amount of nutrients in the soil.

After all this data has been calculated, the farmer still has to decide what this data means, what crops can be planted or what other action can be taken. All this can be achieved with the help of soft computing. Soft computing is a type of computing different from hard computing which is tolerant of imprecision, uncertainty, partial truth, and approximations [3, 4].

This study is based on developing a fuzzy inference system to interpret results of nitrogen, phosphorus, and potassium gotten from soil tests carried on soil samples gotten from southern part of Nigeria (Ibadan) which forms our case study. The results would also be used to predict amount of fertilizer (NPK) that should be applied to the soil to restore its fertility to an optimum level.

The study would help farmers in interpretation of results of their soil test carried out on nitrogen, phosphorus, and potassium. The study is further aimed at better crop and reduction in crop loss from wrong application of fertilizer. The rest of the paper is organized as follows: Literature Review is presented in Section 2 and Methodology is presented in Section 3 while System Implementation is presented in Section 4, and Section 5 comprises Conclusion.

## 2. Literature Review

### 2.1. Soft Computing

There are different definitions of soft computing such as soft computing is a set of “inexact” computing techniques, which are able to model and analyze very complex problems [5]. Soft computing is an emerging approach to computing which parallel the remarkable ability of the human mind to reason and learn in an environment of uncertainty and imprecision [6]. Soft computing is the fusion of methodologies designed to model and enable solutions to real world problems, which are not modeled or too difficult to model mathematically [7]. These methodologies and techniques have been used to create more acceptable cheaper, more complete and approximate solutions to problem. This idea was formulated by Lotfi A. Zadeh in his paper on soft data analysis. The techniques and methodologies include Evolutionary Computing which was developed in 1960 by Rechenberg, Neural Networks developed in 1943 by McCulloch, Fuzzy Logic was developed in 1965 by Lotfi A. Zadeh and Genetic algorithm by John Holland in 1975 which was later popularized by his student David Goldberg [8]. As opposed to hard computing which can also be referred to as conventional computing, which requires a precisely stated analytical model and often a lot of computational time [9], soft computing exploits the tolerance for imprecision, uncertainty, partial truth, and approximation to achieve tractability, robustness, and low solution cost [9]. Fuzzy Logic (FL), Artificial Neural Networks (ANN), and Genetic Algorithms (GA) are considered as core techniques of soft computing [5].

There are have been several applications of soft computing methodologies in different areas of computing such as handwriting recognition [10], Automotive Systems, Manufacturing, Image Processing, Architecture, Decision Support, and Power Systems [9].

#### 2.1.1. Fuzzy Logic

Fuzzy logic is a soft computing technique which uses inexact information to make informed and exact predictions or inference. It takes into account the uncertainty in human decision to make certain predictions and it parallels the remarkable ability of the human mind to reason and learn in an environment of uncertainty and imprecision [11]. The human mind easily reason in an uncertain, in exact way and this manner of reasoning cannot be expressed precisely not even with statistical or probability methods but fuzzy efficiently maps out this uncertainty and inexactness.

Fuzzy set theory was first proposed by Zadeh in 1965 [3, 4] and was first used by Mamdani in control [12] since then, there have been different other applications of fuzzy logic in different fields such as in electronics [13], agriculture, industrial processes, and environmental protection. According to [14],” rather than regard fuzzy theory as a single theory, we should regard the process of “fuzzification” as a methodology to generalize any specific theory from a crisp (discrete) form to a continuous (fuzzy) form”

### 2.2. Application of Fuzzy Logic in Agriculture and Other Fields

Ever since the inception of fuzzy logic in the 1970s, there have been several applications in different fields. Examples of such fields are as follows.

Development of first generation fuzzy logic based hardware [15] provided faster fuzzy control solutions incorporating inference engines. In time past, applications have been based on software modules deployed on conventional microprocessors, personal computers, and workstation type of computing platform. Fuzzy hardware has been implemented in the integration of software algorithms in Integrated Circuits (IC) chips for rule-based fuzzy controllers incorporating fuzzy inference [16].

Some authors have used fuzzy logic to convert heuristic control rules stated by a human operator into an automatic control strategy [17].

Rajesh et al. [18] developed an automated rice cooker with advanced logic technology, which allows it to think for itself and make adjustments to the temperature and timing of cooking of a batch of rice. It made use of a Sugeno style fuzzy approach which used the amount of water, rice, and time as inputs to give the rice cooker the best temperature to cook the rice.

Development of embedded fuzzy applications [19]: a fuzzy logic control of washing machines uses a triangular membership function for the control of washing machines based on specification of the degree of dirt on clothes and it also specifies the amount of soap needed to wash the clothes [20]. Washing machines like this are already in use today.

Fuzzy logic has also been applied in planning of transportation process which is characterized by subjectivity, ambiguity, uncertainty and imprecision [21]. Some Japanese authors also made a crucial addition to fuzzy set theory applications in traffic and transportation [22–24]. Temperature control systems have been extensively designed improved upon using fuzzy logic [25].

There have also been vast applications of fuzzy logic in the area of agriculture such as in the mapping of soil using a combination of GIS, Expert Knowledge, and Fuzzy Logic [26, 27] because standard soil surveys were not designed to provide the detailed (high-resolution) soil information required by some environmental modeling [28].

Reference [29] wrote on development and improvement of an image capture/processing system to distinguish weeds from good plants and a fuzzy logic decision-making system to determine the locations and amount of herbicide to apply in an agrarian field. As data concerning economic thresholds of weed effect on crop profitability cannot easily be adapted to a given area or even to a given farm, a fuzzy logic methodology was applied to convert image data into sprayer commands to permit agriculturists to utilize experience to classify weed status at a given location in the field. This research indicated that a fuzzy logic system has the capability to understand and facilitates the representation and processing of human knowledge in computer and the inputs, outputs, and rules of FL are easy to modify.

Singh and Sharma [30] developed a fuzzy expert system for potato. They recommended optimum amount of NPK fertilizer needed for the soil. The authors made use of three input variables taken nitrogen, phosphorous, and potassium present already in soil which are partitioned into three, four, and two sets, respectively, each. These input variables were represented in triangular shapes. The nitrogen, phosphorous, and potassium needed in the soil accordingly were partitioned into three, four, and two sets each and are represented in triangular shapes. However the portioning into three four and two sets of the input and output variables does not deal effectively with the stratification of the soil.

## 3. Methodology

### 3.1. Data Collection

A soil test was carried out by taking soil samples from different parts of a field. The soil samples collected were mixed together. The sample is thereafter tested in the laboratory and from the result gotten, the levels of nitrogen, phosphorus, and potassium in the soil are then known. All measurements were carried out in parts per million (ppm).

There exist standards of measurement of nitrogen, phosphorus, and potassium in the soil. Kaiumov [31] empirical model stated that there exists an interval of the soil attribute, and if values of this attribute lie within this interval, then its utility is optimal. These tables or standards form the basis for the interpretation of the soil fertility we used in this paper.

### 3.2. Linguistic Variables

From Tables 1 and 2, the linguistic variables for our fuzzy model would be “Low”, “Medium”, “High”, and “Excessive”. We would use them for the membership functions. The Min and Max values would be used as the range for the membership functions of the measured nutrients and the Min and Max values as the range for the membership functions of the required nutrients, respectively.

### 3.3. Membership Functions

The system would make use of triangular membership functions. Therefore they would have a range of three values of the form [Lower limit mid-point Upper limit].

#### 3.3.1. Model of Fuzzy Logic System

In the fuzzy logic system, the input and output variables and their membership functions have to be first decided upon based on the expert's knowledge and experimental studies and a rue base is formed to represent the expert's knowledge. A model is thereafter created in MATLAB based on the input and output variables, their membership functions with their Minimum and Max values, and rules decided upon and transferred to the model.

The rules are checked if they suitably represent the expert's knowledge and are tested if they are correct. If there is any error with any of the rules, the rule is replaced. After all rules have been tested, defuzzification is carried out to produce crisp outputs for the model or system.

After the soil is carried out, the measured nitrogen, phosphorus, and potassium are the input and variable and required nitrogen, phosphorus, and potassium and present level of soil fertility as the output variables and from the experts' knowledge. Fuzzy sets and membership functions are decided upon and fuzzy rules formed. Defuzzification produces crisp values or the outputs.

**Figure 1:**
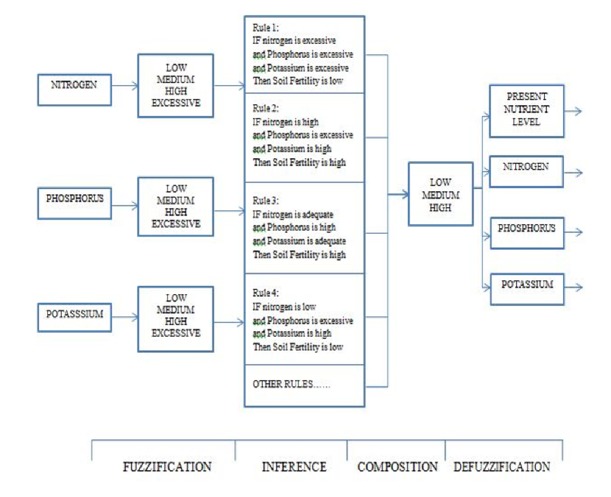
Flowchart of NPK system.

The detailed view of the NPK system is shown in Figure 1 consisting of the input variables, membership functions, rules, and defuzzification process to produce the crisp output. Katorgin (2004), Kaiumov [31], and Singh and Sharma [30] discussed the recommended setting for the values of the mandami fuzzy logic system for input and output for soil fertility. For setting of the values for input in part per million, Table 1 range from 0 to 100 for nitrogen, phosphorus is 0 to 150, while potassium is 0 to 1500 in parts per million. The accepted recommended values for mandami fuzzy logic output settings as illustrated by Kaiumov [31] when converted to parts per million are that when nitrogen is from 0-30 it is categorized as low, 30-70 as average, and 70-100 as high. For phosphorus it is the same as nitrogen while potassium from 0 to 20 is low and 20 to 70 is average while 70 to 100 is high, all in parts per million. The datasets gotten were divided into training and testing. About 70% of the datasets were used for the training while the 30% of the datasets were used for testing to validate the datasets.

### 3.4. Rules

The rules used in this system were based on the following assumptions or postulations culled from the following [32]:

1. When amount of phosphorus is high, phosphorus application is unnecessary and should be limited
2. When phosphorus is low, the rate recommended is intended to satisfy immediate crop needs and begin to produce their own
3. Crops need potassium to be able to use the nitrogen in the soil
4. Even though soil potassium level is optimum, extra is still needed to make up a portion of crop removal
5. The soil can produce its own nitrogen through the use of nitrogen fixing bacteria's and thus does not need extra when its concentration is high

These are the interpretations of different rules that are used:

  H: high
  M: average
  L: low
  E: excessive
  NIL: none
  N: nitrogen
  P: phosphorus
  K: potassium
  R.N: required nitrogen
  R.P: required phosphorus
  R.K: required potassium
  S.F: soil fertility

In this paper, 64 Rules are used with 3 inputs and 4 outputs.

## 4. System lmplementation

### 4.1. Rule Editor

**Figure 2:**
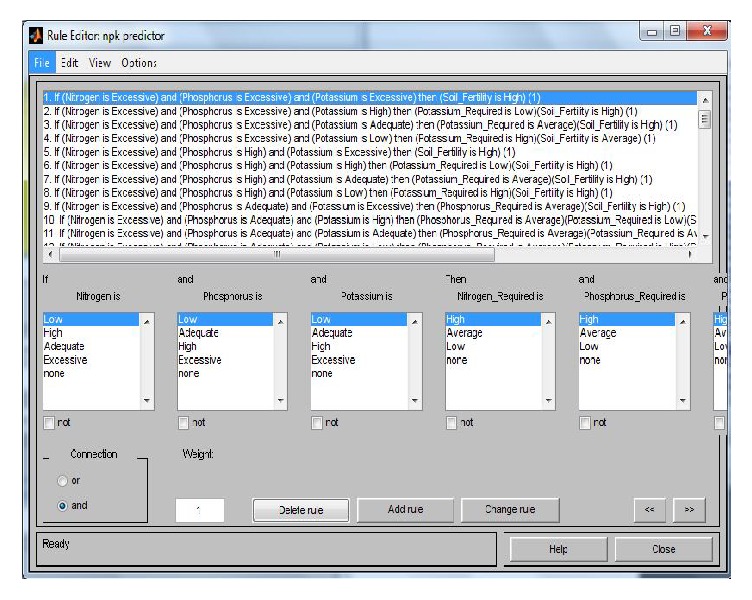
Rule editor.

The rule editor is shown in Figure 2 which hosts the rules conveying the experts' knowledge and binding the inputs together. New rules can be added or existing rules changed by using either of the “Add Rule” or “Change Rule” buttons, respectively, and a rule can be deleted using the “Delete Rule" button. The rules are joined together using the AND or OR connective.

The rule viewer allows the user to see the effect of each input on the outputs based on the rules inputted. It gives the user the defuzzified results.

### 4.2. Program Implementation

**Figure 3:**
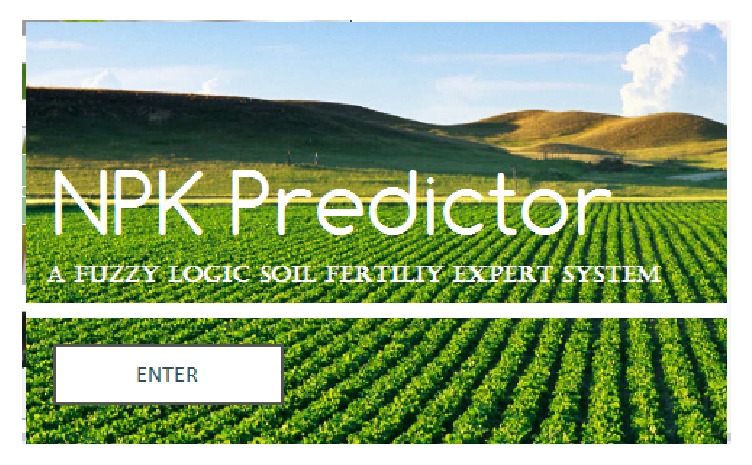
Front page.

Figure 3 shows the front page that the user gets to on starting the application. It contains the name of the application and a short description about it. Upon clicking the enter button, the user is taken to a new page or interface for entering the values of each of the components.

### 4.3. Reading Dialog Interface

**Figure 4:**
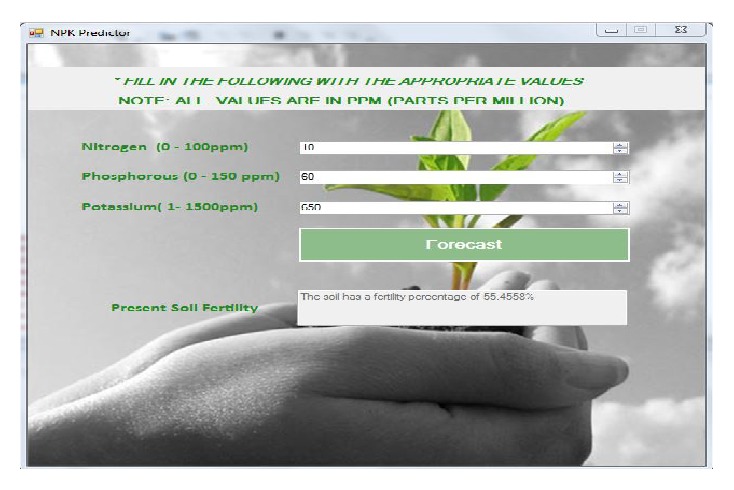
Reading dialog interface showing a user's input.

**Figure 5:**
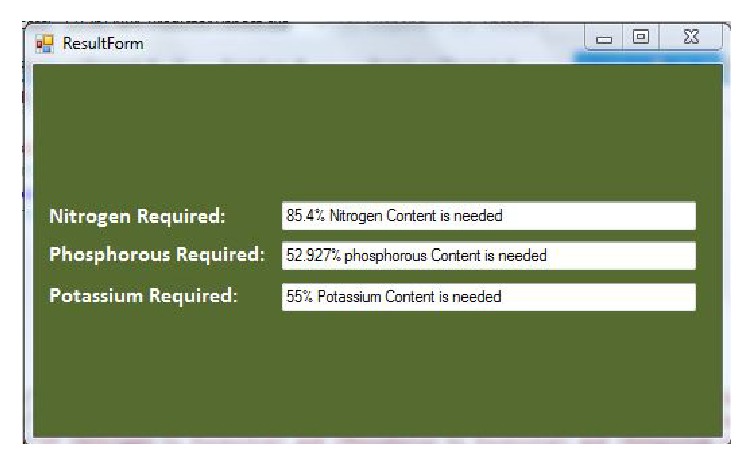
Reading dialog interface showing the result of a user's input in Figure 4.

Figure 4 allows the user to be able to enter the nitrogen, phosphorus, and potassium results gotten from the soil test he has carried out on his farm. The values added are in PPM (parts per million). After entering the values, the user clicks on the forecast button and he receives the present level of soil fertility based on the values entered in percentage and thereafter, the user gets recommended values of NPK needed for the fertility of the soil to reach an optimum level as shown in Figure 5. The recommended values given to the user are also in PPM and the user can then go ahead to use it to calculate the amount of fertilizer needed in the soil.

### 4.4. Prediction Accuracy of the Proposed System

The prediction accuracy of the proposed system was carried out and compared with the existing system commonly known as J48 algorithm for predicting soil fertility. We used the formulae (1), (2), and (3):

(1) TPR=TPP

(2) TNR=TNN

where TPR is true positive rate, TP = true positive and P= positive, TNR = true negative rate, TN = true negative, and N= negative.

(3) $$\begin{array}{c} False\;\;Alarm\;\;Rate=\frac{Number\;\;of\;\;Major\;\;Deviations}{Total\;\;Number\;\;of\;\;Data} \end{array}$$

From Table 3, generally the results gotten from our work showed that fuzzy logic outperformed the J48 in predicting soil fertility. 1.2% and 1.7% false alarms were discovered in fuzzy logic and J48 method, respectively. Using formulae (1), (2) and (3) above, the percentage of predicting accuracies were found to be apparently better than the results gotten from other method of J48 algorithm for predicting soil fertility. The datasets used for this work were partitioned into two. 70% of the datasets collected were used for the training while the remaining 30% of the datasets were used for testing to validate the datasets.

## 5. Summary, Recommendation, and Conclusion

### 5.1. Summary

This paper has shown how fuzzy logic as an expert system can be applied to the forecasting of soil fertility. The modularity of the system enables the verification of the rules used in the system. The separation of control from knowledge would allow for expansion of the knowledgebase of the system without affecting the total functionality of the system. The use of fuzzy logic also takes into account the variability and imprecision of soil test readings. The system can work on any windows enabled workstation.

### 5.2. Recommendation

The project recommends for further work by the use of Artificial Neuro Fuzzy Inference System (ANFIS) to analyze the system and also due to the different nutrients demands of different crops, crop types can be included as criteria for nutrient recommendation in future studies.

### 5.3. Conclusion

The application of this concept of the fuzzy expert system approach would proffer a better soil quality which would invariably offer a means to assess soil quality as "a degree or grade of perfection".

Fuzzy logic has continued to gain grounds since its inception and there are more and more applications of fuzzy logic that have been developed. There is still a lot to be done in the agricultural sector and this study believes that as our knowledge of fuzzy logic keeps increasing it would be applied to other sectors.

## Figures and Tables

**Table 1:** Input variables with their linguistic functions showing minimum & maximum values.

INPUT	LEVEL	MIN VALUE	MAX VALUE
NITROGEN	LOW	-1	20
	ADEQUATE	20.1	41
	HIGH	41.1	70
	EXCESSIVE	70.1	100

PHOSPHORUS	LOW	-1	20
	ADEQUATE	20.1	40
	HIGH	40.1	100
	EXCESSIVE	100.1	150

POTASSIUM	LOW	0	150
	ADEQUATE	150.1	250
	HIGH	250.1	800
	EXCESSIVE	800.1	1500

**Table 2:** Output variables showing linguistic variables and recommended values.

OUTPUT	LEVEL	RECOMMENEDED VALUE
NITROGEN	LOW	0 - 30
	AVERAGE	30 - 70
	HIGH	70 - 100

PHOSPHORUS	LOW	0 - 30
	AVERAGE	30 - 70
	HIGH	70 - 100

POTASSIUM	LOW	0 - 20
	AVERAGE	20 - 70
	HIGH	70 - 100

SOIL FERTILITY	LOW	0 – 40
	AVERAGE	40 – 80
	HIGH	80 - 100

**Table 3:** Predictive accuracies of fuzzy logic with J48 for soil fertility.

Prediction Accuracy	J48 Algorithm	False Positive
J48	87.6%	1.7

Fuzzy Logic	99.1%	1.2

## Data Availability

The data used to support the findings of this study are available from the corresponding author upon request.
